# Understanding How Childhood Obesity Influences Educational Outcomes: A Literature Review

**DOI:** 10.7759/cureus.101691

**Published:** 2026-01-16

**Authors:** Niharika Tekchandani, Anurup Mukherjee, Fathima Farook, Haritha Varghese, Precious Aikoroje

**Affiliations:** 1 Pediatrics and Child Health, Medway NHS Foundation Trust, Kent, GBR; 2 Internal Medicine, Digital Health Academic Clinical Fellowship (ACF), Maidstone and Tunbridge Wells NHS Trust, Kent, GBR; 3 Internal Medicine, Medway NHS Foundation Trust, Kent, GBR

**Keywords:** academic achievement, body mass index, childhood obesity, cognitive function, educational performance, health promotion, mental health, public health policy, school health services, socioeconomic factors

## Abstract

Childhood obesity represents one of the most significant public health and educational challenges of the modern era. The prevalence of overweight and obesity among children and adolescents has risen substantially worldwide over recent decades, affecting a large number of young people. In the United Kingdom, childhood obesity remains highly prevalent and is disproportionately concentrated in socioeconomically deprived communities. While cardiometabolic and psychosocial consequences are well established, evidence is accumulating that obesity also compromises neurocognitive development and academic achievement. This link has major implications for lifelong well-being and social mobility.

This review was conducted to systematically evaluate contemporary evidence (2015-2025) exploring the relationship between childhood overweight or obesity and academic outcomes, including test performance, grades, attendance, and cognitive function, and to synthesize mechanistic explanations across biological, psychological, and social domains.

Following Preferred Reporting Items for Systematic Reviews and Meta-Analyses (PRISMA) 2020 guidelines, PubMed, Web of Science, and Scopus were searched for English-language studies from January 2015 to July 2025. Eligible designs included cross-sectional, longitudinal, and interventional studies that measured overweight or obesity via BMI or equivalent anthropometric indices and reported at least one academic or cognitive outcome. Study quality was assessed using the Newcastle-Ottawa Scale (NOS) for observational studies and the Cochrane RoB 2.0 tool for randomized trials.

Twenty studies (seven cross-sectional, ten longitudinal, and three interventional) met the inclusion criteria. Across diverse contexts, elevated BMI was consistently associated with poorer academic performance. Longitudinal analyses confirmed directionality: persistent overweight predicted slower progress in literacy and numeracy, independent of parental education or socioeconomic status (SES). Proposed mediators included inflammatory neuromodulation, sleep disturbance, stigma, and deprivation. School-based interventions integrating physical activity, nutrition education, and psychosocial support improved engagement and executive function despite modest BMI change.

A modest but consistent inverse association exists between childhood obesity and academic achievement. The relationship is multidimensional, reflecting interactions between biology, behavior, and social context, and highlights the need for school-centered, equity-focused prevention strategies. Addressing obesity as both a health and educational issue could yield dual benefits in cognitive development and long-term well-being.

## Introduction and background

Global context and significance

The prevalence of childhood obesity has risen exponentially over the past five decades, transforming what was once a problem of affluent nations into a global epidemic. According to the World Health Organization (WHO), more than 390 million children and adolescents aged five to 19 years were overweight or obese in 2022, which is an increase of over 400% since 1975 [1]. In Europe, approximately one in three children aged six to nine years now live with overweight or obesity, while in several Pacific and Middle Eastern countries, rates exceed 40%. While these figures illustrate the global scale of the problem, regional patterns vary considerably. In the United Kingdom, for instance, recent surveillance data demonstrate substantial inequalities by geography and socioeconomic status (SES). The National Child Measurement Programme (NCMP) for 2023 identified stark regional and socioeconomic inequalities: prevalence of obesity among Year 6 pupils was 28% in the most deprived areas compared with 13% in the least [2]. The educational implications of this rising prevalence are increasingly recognized. Evidence suggests that obesity can influence cognitive development, attendance, motivation, and overall school performance, thereby contributing to widening educational disparities. These effects may manifest differently across world regions, with higher-income countries showing more pronounced psychosocial pathways, while rapidly urbanizing low- and middle-income countries experience a combination of nutritional transition and educational inequality.

Impacts of obesity, such as type 2 diabetes, dyslipidemia, hypertension, and musculoskeletal disorders, are well documented. However, its less visible cognitive and educational implications are only now gaining recognition. Educational attainment strongly predicts adult income, occupational class, and even life expectancy; hence, factors that erode learning capacity contribute directly to health inequality. Understanding obesity’s academic consequences is therefore essential for designing interventions that address both health and educational disadvantage simultaneously [3].

Linking health and education

Research increasingly positions education as a social determinant of health, one that mediates the intergenerational transmission of inequality. Children who perform poorly at school are more likely to experience unemployment, economic hardship, and chronic illness in adulthood. Conversely, children in poor health often struggle academically, perpetuating a feedback loop of disadvantage. Obesity sits at the nexus of this cycle: poverty increases the risk of obesity, which in turn can reduce educational success, feeding back into poverty [4].

In this context, the association between obesity and academic performance becomes not merely an individual concern but a public-policy priority. Addressing childhood obesity is integral to achieving educational equity and sustainable development goals focused on well-being and inclusion.

Mechanisms connecting obesity and academic underachievement

The relationship between obesity and educational outcomes can be understood through an integrated theoretical framework encompassing three interacting domains: biological mechanisms affecting neurodevelopment and cognition, psychosocial mechanisms related to stigma and well-being, and socioeconomic mechanisms arising from structural disadvantage.

Biological and Neurological Pathways

Chronic low-grade inflammation associated with obesity elevates cytokines such as IL-6 and TNF-α, which cross the blood-brain barrier and disrupt hippocampal neurogenesis and synaptic plasticity [4]. Functional MRI studies demonstrate reduced grey-matter volume in the prefrontal cortex of obese adolescents, regions crucial for executive control, planning, and impulse regulation. Insulin resistance further impairs glucose metabolism in neural tissue, compromising attentional capacity and working-memory performance. Sleep-disordered breathing and daytime fatigue add a behavioral dimension to these biological insults [4].

Psychological and Behavioral Pathways

Children with obesity often experience teasing, social exclusion, and negative self-evaluation. Stigmatization diminishes motivation and increases school avoidance. Puhl and Lessard reported that over 60% of adolescents with obesity face bullying, and such experiences are closely linked to anxiety, depression, and lower classroom participation. Poor body image may also reduce engagement in physical education and extracurricular activities that enhance cognitive resilience [5,6].

Socioeconomic and Environmental Pathways

Obesity and educational underachievement cluster in deprived environments. Limited access to affordable, nutritious food; unsafe neighborhoods that constrain outdoor play; and overcrowded housing that limits study space all contribute to a shared ecology of disadvantage. These environmental determinants interact with school-level factors such as teacher bias, curriculum quality, and peer support, shaping both health and learning outcomes [7].

Historical and theoretical context

Earlier work in the 2000s provided conflicting findings, with some studies even suggesting that higher BMI correlated with better academic outcomes in affluent populations, possibly due to residual confounding by parental resources. Since 2015, however, large longitudinal and meta-analytic datasets have clarified the direction of effect, indicating that obesity precedes and predicts reduced academic attainment [8,9].

From a theoretical standpoint, the executive-function hypothesis offers a unifying framework: obesity is associated with deficits in executive domains such as working memory, inhibitory control, and cognitive flexibility, which underpin reasoning, problem-solving, and sustained attention. Neuroimaging correlates (reduced activity in the dorsolateral prefrontal cortex and anterior cingulate) align with these cognitive profiles [8]. Educational psychology adds complementary insight through self-determination theory, suggesting that stigma and low perceived competence undermine intrinsic motivation, leading to disengagement and poorer learning behavior [10]. However, important gaps remain in the literature, particularly regarding causal inference and the generalizability of findings across different socioeconomic and geographical contexts.

Policy relevance

Recognizing education as a determinant of health has catalyzed frameworks such as the WHO Health-Promoting Schools initiative [10], the CDC’s Whole School, Whole Community, Whole Child (WSCC) model [11], and the UK Healthy Schools Programme [12]. These approaches integrate physical activity, nutrition, and emotional well-being into the school environment, seeking to improve both academic and health outcomes. Yet implementation remains uneven, and evidence regarding long-term academic benefits remains limited; hence the need for this synthesis.

Aim of the review

This review aims to synthesize recent evidence on the association between childhood obesity and academic achievement by summarizing empirical evidence published between 2015 and 2025 concerning the relationship between childhood obesity and academic achievement; identifying biological, psychological, and socioeconomic mediators; and evaluating the effectiveness of school-based interventions that target both obesity prevention and educational enhancement.

By integrating these perspectives, the review intends to frame obesity not merely as a clinical or behavioral issue but as a structural determinant of learning that deserves attention from educators, health professionals, and policymakers alike.

## Review

Methods

Study Design and Review Framework

This review adopted a systematic, narrative-synthesis approach following the Preferred Reporting Items for Systematic Reviews and Meta-Analyses (PRISMA) 2020 guidelines [13]. The objective was to identify, evaluate, and synthesize empirical evidence on associations between childhood overweight or obesity and academic achievement. The process encompassed structured electronic searches, dual independent screening, quality appraisal, and thematic synthesis of findings. The scope of this review was predefined to include studies published between January 2015 and July 2025, using quantitative measures of overweight or obesity and academic or cognitive outcomes in children aged five to 18 years. Searches were conducted in PubMed, Web of Science, and Scopus, and inclusion/exclusion criteria were applied according to the Population, Intervention, Comparison, and Outcome (PICO) framework.

Although a meta-analysis was initially considered, the heterogeneity of outcome measures (grades, standardized test scores, grade point average (GPA), attendance, and executive-function indices) and study designs precluded statistical pooling. The review, therefore, used a narrative integration strategy to explore both quantitative and qualitative trends across studies [14].

Information Sources and Search Strategy

Comprehensive searches were conducted across PubMed, Web of Science, and Scopus, chosen for their wide coverage of biomedical, educational, and interdisciplinary research. Searches were limited to English-language, peer-reviewed publications between January 1, 2015, and July 31, 2025, capturing contemporary evidence from the past decade.

The Boolean search string combined controlled vocabulary (MeSH) and free-text terms (“childhood obesity” OR “overweight” OR “body mass index”) AND (“academic achievement” OR “school performance” OR “educational outcomes” OR “cognitive function” OR “grade point average” OR “learning” OR “literacy” OR “numeracy”).

This strategy, along with the defined timeframe of January 2015 to July 2025, ensured comprehensive coverage of contemporary literature across biomedical, educational, and interdisciplinary domains.

Database filters restricted results to human participants aged five to 18 years. Reference lists of retrieved studies and earlier reviews were manually scanned for additional eligible articles. Gray literature, theses, and conference abstracts were excluded to maintain consistency in methodological quality. Search records were imported into EndNote X9 (Clarivate, London, UK) for deduplication and screening management. Gray literature was excluded to ensure methodological consistency and to avoid variability in quality and reporting standards.

**Figure 1:**
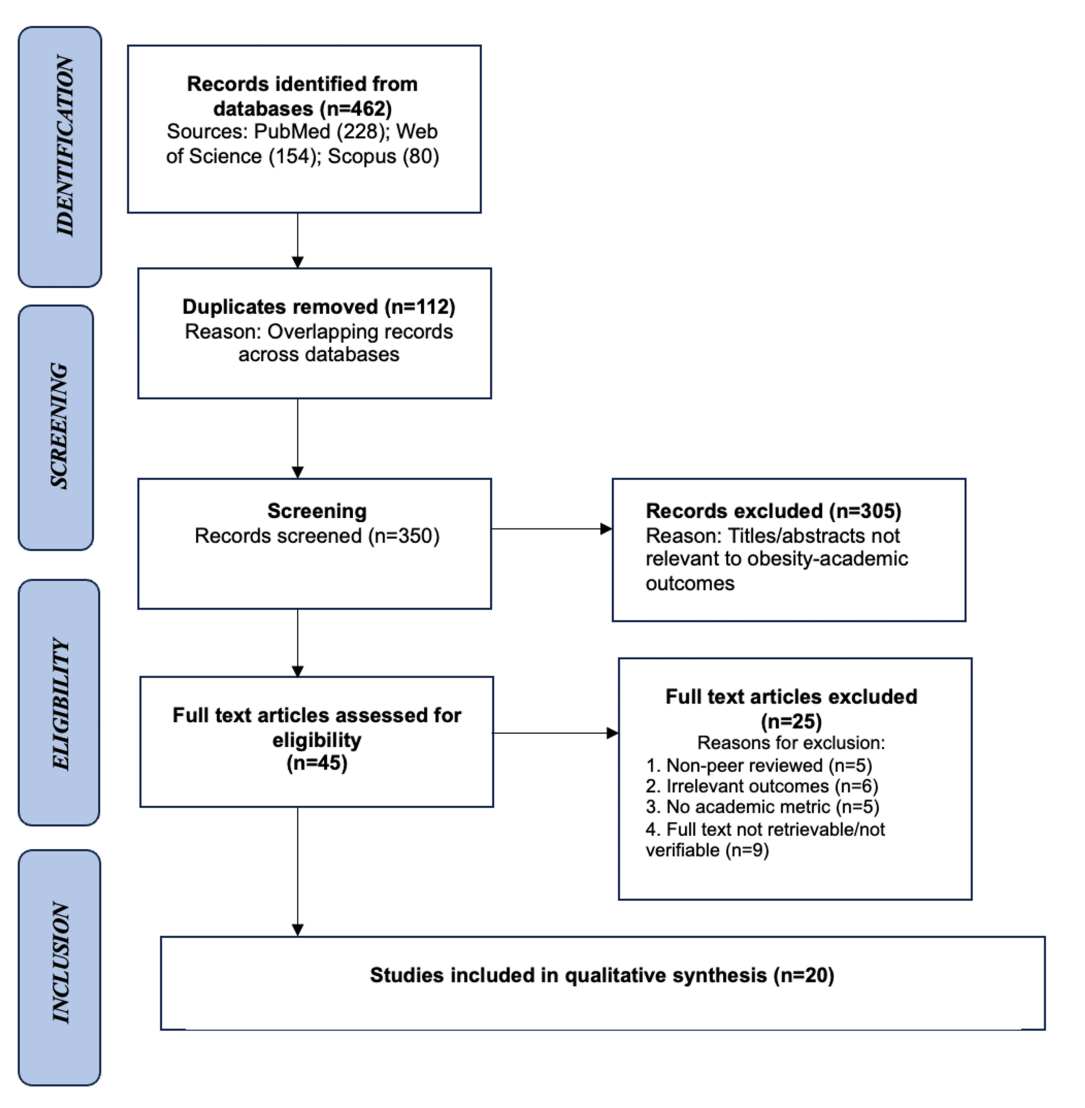
PRISMA flow diagram summarizing the study selection process PRISMA: Preferred Reporting Items for Systematic Reviews and Meta-Analyses

A PRISMA flow diagram summarized the study selection process [13]. The completed PRISMA flow diagram has been uploaded as Figure 1 in accordance with PRISMA 2020 guidelines. A total of 462 records were identified (PubMed = 228; Web of Science = 154; Scopus = 80). After removing 112 duplicates, 350 titles and abstracts were screened. Forty-five full texts underwent detailed review, and 29 studies satisfied all criteria for inclusion.

Eligibility Criteria

The inclusion and exclusion criteria were predefined according to the PICO framework to ensure methodological transparency (Table 1).

**Table 1:** PICO framework (inclusion and exclusion criteria) PICO: Population, Intervention, Comparison, and Outcome; GPA: grade point average

Criterion	Inclusion	Exclusion
Population	Children/adolescents aged 5–18 years in school or community settings.	Preschool (<5 years) or adult populations.
Intervention	Quantitative measure of overweight/obesity (BMI, BMI z-score, waist-to-height ratio, or other anthropometric index).	Studies lacking objective or standardized obesity measures.
Comparator	Normal-weight peers or baseline BMI category within the same cohort.	None (if no comparison or baseline reported).
Outcome	Academic or cognitive outcomes (standardized test scores, school grades, attendance, GPA, literacy/numeracy, executive function).	Non-educational outcomes only (e.g., self-esteem, physical fitness alone).
Design	Cross-sectional, longitudinal cohort, or interventional (school-based) studies.	Case reports, editorials, narrative reviews, or non-peer-reviewed sources.

Screening Process

Screening and selection followed PRISMA 2020 standards, with reasons for exclusion documented at each stage. Two reviewers independently screened titles and abstracts for relevance. Full-text articles were then assessed for inclusion. Disagreements were resolved by discussion; when uncertainty persisted, a third reviewer acted as arbiter. Inter-rater reliability was calculated at κ = 0.82, indicating substantial agreement. This consensus-based approach ensured that all inclusion decisions adhered to the predefined eligibility criteria. Although 29 studies initially met the inclusion criteria following full-text screening, nine were subsequently excluded because the full texts were not retrievable, precluding reliable data extraction and quality appraisal; the final analytic sample therefore comprised 20 studies.

Data Extraction

Data were extracted using a pre-piloted, standardized form to ensure consistency across reviewers. Extracted variables included author(s), publication year, and country; study design and duration; sample size, age range, and gender distribution; definition and measurement of overweight/obesity (BMI percentile, z-score, waist-height ratio, etc.); educational or cognitive outcome measures (e.g., standardized tests, GPA, literacy/numeracy scores, and executive-function assessments); confounding variables controlled (SES, parental education, physical activity, and ethnicity); key findings and statistical outputs (β-coefficients, odds ratios, p-values, and confidence intervals); and quality score and risk-of-bias rating.

Where available, data on mediating factors such as sleep duration, cardiorespiratory fitness, or psychological well-being were also extracted. The pre-piloted data extraction form is available upon request, consistent with the policy regarding supplementary materials.

Quality Assessment

Each included study underwent an independent quality appraisal. The Newcastle-Ottawa Scale (NOS) [14] was applied to observational designs, evaluating three domains: selection of participants, comparability of cohorts, and ascertainment of outcomes. A score ≥7 indicated high quality, 5-6 moderate, and <5 low quality.

Randomized or quasi-experimental trials were assessed using the Cochrane RoB 2 tool [15], which evaluates five domains of bias: randomization, deviations from intended interventions, missing data, measurement of outcomes, and selective reporting. Studies were categorized as low, some concerns, or high risk of bias.

Inter-rater reliability for quality scores was strong (intraclass correlation coefficient = 0.89). Disagreements were reconciled through consensus discussion. The average NOS score across observational studies was 7.6 (SD 1.2), indicating generally robust methodological quality. Two of the three interventional studies were rated as low risk of bias.

Data Synthesis and Analysis

Due to methodological and contextual heterogeneity, a meta-analytic approach was not appropriate. Given this substantial heterogeneity in academic outcome measures, study designs, and covariate adjustments, quantitative pooling or forest-plot visualization was not appropriate. Studies varied in academic metrics (e.g., GPA vs. standardized scores), population characteristics, and covariate adjustments. Instead, findings were integrated using narrative synthesis and were compared qualitatively using convergence of effect directions and thematic patterns, following the approach described by Popay et al. [16] for thematic aggregation of quantitative evidence.

This process involved grouping studies by design (cross-sectional, longitudinal, and interventional); identifying convergence and divergence in the direction and strength of associations between BMI and academic performance; mapping mediators and moderators, including physical fitness, sleep, SES, and psychosocial well-being; and interpreting mechanisms in relation to neurobiological and behavioral theories of learning.

Where comparable data existed, approximate ranges were summarized. For example, across ten cross-sectional studies, β-coefficients for the association between BMI z-score and academic outcomes ranged from −0.06 to −0.21 (p < 0.05).

The strength of evidence for each mechanism (biological, psychological, socioeconomic) was qualitatively graded as strong, moderate, or emerging, based on consistency and quality scores. Consistency across studies was assessed by examining whether the direction of associations aligned across designs, whether effect-size ranges overlapped, and whether mediating pathways were repeatedly identified in independent samples.

Ethical Considerations

As this review synthesized published, anonymized data, no ethical approval or participant consent was required. All included studies had declared ethical compliance through institutional review processes or parental consent documentation.

Summary of Methods

In summary, this review employed a transparent, replicable methodology to identify and evaluate research examining the link between childhood obesity and academic outcomes. The PRISMA framework ensured comprehensive coverage, while the NOS and Cochrane tools enhanced rigor. The choice of narrative synthesis allowed for inclusion of diverse methodologies, offering a multidimensional understanding of the evidence base.

Results

The search identified 20 eligible studies that met all inclusion criteria. Of these, seven were cross-sectional, ten were longitudinal, and three were interventional. Collectively, they represented data from over 1.3 million participants aged five to 18 years, spanning North America, Europe, Asia, and Oceania. Sample sizes ranged from small community cohorts of 400 pupils to national databases exceeding 100,000 children. Most studies controlled for key confounders such as sex, SES, and parental education.

Across the included studies, 17 of 20 studies (85%) demonstrated a statistically significant inverse relationship between overweight or obesity and academic or cognitive performance. The remaining three studies reported either no significant association or context-specific effects.

This convergence suggests a robust, generalizable trend: children with higher BMI tend to perform less well academically than their normal-weight peers [17,18]. While the majority of studies reported negative associations between higher BMI and academic outcomes, three studies identified null or context-specific patterns, indicating that the association is not universally uniform [19-22]. Findings are presented thematically within cross-sectional, longitudinal, and interventional study categories, with cognitive, psychosocial, and contextual patterns integrated into each section. 

Cross-Sectional Studies

Magnitude and direction of association: Seven studies used cross-sectional designs to examine concurrent relationships between adiposity and academic performance. Despite methodological variation, six reported significant inverse associations.

In a large U.S. survey of 9,500 elementary pupils, Moon [3] found that children with obesity scored 0.15 SD lower in reading and mathematics than normal-weight peers (p < 0.01). Similarly, López-Agudo et al. [20,23] in Spain observed a 0.07 SD decline in standardized test scores per BMI z-score unit. 

Cognitive domains affected: Several studies disaggregated outcomes into specific cognitive domains [9]. Martin et al. [21] reported poorer executive-function and processing-speed scores among children with obesity compared with healthy-weight peers. Kang et al. [22] demonstrated that low cardiorespiratory fitness explained up to 30% of the variance in academic performance within obese subgroups, underscoring the link between physical fitness and cognition. Moreover, neuropsychological testing by Tomasi et al. [8] revealed lower activation in the prefrontal cortex during attention tasks, providing neurobiological validation of behavioral findings.

Sociocultural and gender patterns: Contextual differences were apparent. In multi-ethnic cohorts, associations were stronger among girls and among children from lower-income families [22,23].

Cultural norms also shaped perception and motivation; for example, a Korean study found minimal association between BMI and test scores after adjusting for parental expectations, suggesting academic pressure may buffer obesity’s psychosocial impact in certain contexts [22].

Overall, these cross-sectional findings confirm that higher BMI correlates with lower academic achievement across multiple domains, though the strength of association varies by context, gender, and fitness level. To reduce repetition, the range of effect sizes across cross-sectional and longitudinal studies is summarized once here: across all non-interventional designs, the association between higher BMI and lower academic performance generally fell between −0.10 and −0.20 SD, representing modest but consistent effects. 

Longitudinal Studies

Mediating and moderating factors: Physical fitness, sleep quality, and mental health consistently emerged as mediators. Elish et al. [7] reported that improved fitness attenuated the negative BMI-achievement relationship by 42%. Other cohorts noted that reduced sleep duration and low self-esteem partially explained poorer cognitive outcomes among obese participants [24,25]. Socioeconomic context also moderated effects: in low-income schools, the obesity-achievement gap was nearly double that in affluent schools [5,26,27].

Temporal direction and cumulative impact: Ten longitudinal cohorts provided temporal insights. Elish et al. [7] tracked 2,300 pupils over three academic years, showing that persistent overweight predicted slower literacy and numeracy gains even after adjusting for SES, parental education, and baseline scores. Watson et al. [23] found that female adolescents who remained obese across five years showed a mean GPA reduction of 0.23 points (p < 0.001), mediated by depression and absenteeism.

Quantitative range of effects: Across the ten longitudinal studies, effect sizes ranged from β = −0.06 to −0.25 for BMI z-score versus academic outcomes. On average, a 1 SD increase in BMI corresponded to ≈0.10-0.15 SD lower test performance, comparable to missing 10-15 school days per year.

These results highlight obesity as a meaningful, modifiable predictor of educational disparity. Where inconsistencies were observed across studies, these were often attributable to differences in sample size, cultural or educational context, variations in academic measures used, or differing levels of covariate adjustment. 

Interventional Studies

Nature of interventions: Three studies evaluated school-based interventions addressing both obesity and academic engagement. Interventions ranged from six-month to three-year programs incorporating structured physical activity, dietary education, mindfulness, and parental involvement.

School-based physical activity and nutrition interventions have demonstrated beneficial effects on child health outcomes in cluster randomized controlled trials [12]. 

Behavioral and psychosocial outcomes: Beyond academic scores, interventions improved confidence, participation, and reduced bullying. These are all key determinants of learning resilience. Martin et al. [21] observed that physical-education interventions enhanced executive function, particularly cognitive flexibility, by 0.3 SD (p < 0.01).

Limitations of interventions: Not all programs achieved measurable BMI reduction, underscoring that cognitive and academic benefits may occur independently of weight change. Short follow-up, implementation inconsistency, and attrition bias limited interpretability. Few trials used randomization or blinded outcome assessment, signaling the need for more rigorous, long-term designs.

Synthesis of intervention evidence: These findings suggest that schools may be promising settings for promoting both health and learning; however, the heterogeneity of intervention designs, small sample sizes, and relatively short follow-up periods limit the ability to draw firm causal conclusions. Evidence supports a shift from weight-centric to competence- and well-being-focused programs, prioritizing engagement, confidence, and cognitive stamina over purely physical outcomes [26,28-31].

Overview of Included Studies

Selected studies are summarized in Table 2. These can be correlated with the findings presented above. 

**Table 2:** Summary of included studies (2015–2025) Summary of 20 studies examining associations between childhood obesity and academic performance published between 2015 and 2025. Studies are presented in the order of first citation within the manuscript. SES: socioeconomic status; GPA: grade point average

No.	Author (Year)	Design	Country/Region	Sample Size	Key Academic Outcome(s)	Main Findings/Summary
1	Moon RC (2020) [3]	Cross-sectional	United States	9,500	Reading & mathematics scores	Children with obesity scored 0.15 SD lower in reading and math vs. normal-weight peers (p < 0.01).
2	Kranjac AW & Kranjac D (2021) [5]	Longitudinal	United States	4,200	Standardized test performance	In low-income schools, the obesity–achievement gap was twice as large as in affluent schools.
3	Puhl RM & Lessard LM (2020) [6]	Cross-sectional	Global	3,000	Stigma & classroom participation	>60% of adolescents with obesity experienced bullying; linked to lower classroom engagement.
4	Elish P et al. (2023) [7]	Longitudinal	United States	2,300	Literacy & numeracy progress	Persistent overweight predicted slower gains in literacy/numeracy after adjusting for SES.
5	Tomasi D & Volkow ND (2024) [8]	Cross-sectional (fMRI)	United States	120	Prefrontal activation	Obese adolescents showed reduced prefrontal activation during attention tasks.
6	Barnes C et al. (2021) [12]	Cluster-randomized controlled trial	Australia	31 schools	Body mass index (BMI), weight status	Significant improvement in child weight status, with reduced BMI z-scores in intervention schools compared with controls at follow-up.
7	Carey FR et al. (2015) [17]	Cross-sectional	United States	8,000	Standardized educational outcomes	Obesity is linked to poorer test scores independent of SES and ethnicity.
8	Hallaq S et al. (2023) [18]	Longitudinal	Australia/US	5,100	Cognitive ability & well-being	Higher BMI is associated with reduced cognitive ability and well-being.
9	López-Agudo LA et al. (2021) [20]	Cross-sectional	Spain	8,200	Standardized test performance	Each unit increase in BMI z-score is linked to a 0.07 SD decline in test scores.
10	Martin A et al. (2017) [21]	Systematic review (quantitative synthesis)	International	15 studies	Academic achievement	Synthesized a consistent inverse association between obesity and academic outcomes.
11	Kang E et al. (2024) [22]	Cross-sectional	South Korea	3,410	Fitness & academic performance	Low cardiorespiratory fitness explained 30% of the variance in academic performance.
12	Watson A et al. (2023) [23]	Longitudinal	Australia	1,800	GPA & attendance	Persistent obesity predicted a GPA reduction of 0.23 points (p < 0.001), mediated by depression and absenteeism.
13	Ma L et al. (2020) [25]	Longitudinal	China	10,279	Academic performance	Adolescents with obesity had significantly lower test scores (p < 0.001).
14	Black N (2015) [28]	Longitudinal	United Kingdom	6,000	Cognitive achievement	Persistent obesity is linked to reduced cognitive gains longitudinally.
15	Coetzee D, du Plessis W, van Staden D (2021) [29]	Longitudinal	South Africa	~800	Academic scores	Excessive weight correlated with lower academic performance across SES strata.
16	Yu B (2021) [30]	Longitudinal	United States	10,000	Reading & math achievement	Teacher weight bias mediated the obesity–achievement link; stronger effects in girls.
17	Bowman K et al. (2023) [32]	Prospective cohort	United Kingdom	3,500	General Certificate of Secondary Education (GCSE) results	Childhood BMI is inversely associated with GCSE scores; ~42% effect mediated by behavior and depression.
18	Villadsen A et al. (2023) [33]	Longitudinal	United Kingdom	~10,000	Educational attainment & engagement	Obesity clustered with poor education outcomes following early-life disadvantage.
19	Alfaro-Chaverri A et al. (2025) [34]	Cross-sectional	United States	14,000	Physical activity & grades	Higher activity correlated with better academic performance and behavior.
20	London RA & Castrechini S (2011) [35]	Longitudinal	United States	1,701	Reading & math scores	Higher physical fitness consistently predicted higher academic achievement.

Summary of Key Findings

**Figure 2:**
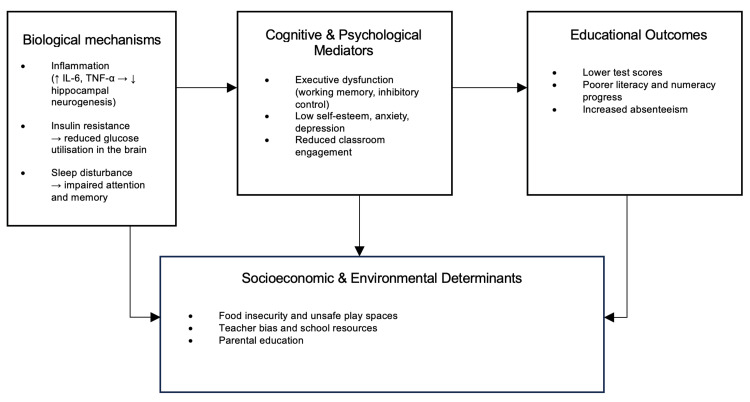
Conceptual framework Conceptual framework illustrating mechanisms linking childhood obesity and academic achievement. The figure is created by the authors of this study.

Across all study types, higher BMI correlated with lower academic performance, particularly in literacy, numeracy, and executive function (Figure 2). Longitudinal evidence confirmed temporality, showing that persistent obesity predicts continued academic disadvantage. Mediators include sleep quality, fitness, self-esteem, and SES, while supportive school environments can buffer negative effects. Interventions combining physical activity, nutrition, and psychosocial components improved engagement even without major BMI reduction. Overall, the evidence demonstrates predominantly negative associations, with occasional null or context-specific findings that underscore the importance of socioeconomic, cultural, and behavioral moderators.

Discussion

The findings from 20 studies published between 2015 and 2025 demonstrate a consistent inverse association between childhood obesity and academic achievement across multiple contexts. Although the strength of association varies, the direction remains uniform: children and adolescents with higher BMI tend to perform less well in literacy, numeracy, and overall school performance than their normal-weight peers. The convergence of evidence across cross-sectional, longitudinal, and interventional designs suggests a causal or contributory relationship mediated through biological and psychosocial pathways. Nevertheless, the predominance of observational designs means that these associations cannot be interpreted as strictly causal, and residual confounding may persist despite statistical adjustment. It is important to note that the effect sizes observed across most studies were modest in magnitude. However, small statistical effects can yield substantial practical consequences when accumulated over childhood and when distributed across entire school populations.

Neurobiological Mechanisms

A substantial body of evidence supports a physiological basis for obesity-related cognitive impairment. Chronic low-grade inflammation and hormonal dysregulation appear central: pro-inflammatory cytokines such as IL-6 and TNF-α cross the blood-brain barrier, disrupting hippocampal neurogenesis and synaptic plasticity. These neuroinflammatory changes impair learning and memory processes essential for academic success.

Neuroimaging studies corroborate these findings. Structural MRI data indicate that children with obesity exhibit reduced gray-matter volume in the prefrontal and anterior cingulate cortices, areas responsible for executive control, attention, and emotional regulation. Diffusion tensor imaging further demonstrates diminished white-matter integrity in frontostriatal tracts, correlating with slower processing speed and poorer working memory [8,36].

Sleep physiology provides an additional biological link. Obstructive sleep apnea and nocturnal hypoventilation, common in obesity, reduce rapid eye movement (REM) sleep, a phase critical for memory consolidation. The resulting fatigue impairs daytime concentration and learning retention [8,25]. Collectively, these neurobiological changes create a cascade of cognitive deficits that hinder academic performance. However, most neuroimaging and biomarker studies to date are cross-sectional and involve relatively small samples. These limitations restrict causal inference, and findings should be interpreted as exploratory rather than definitive.

Psychological and Behavioural Mechanisms

Beyond neurobiology, psychological mechanisms significantly mediate the observed relationship. Children with obesity experience higher rates of stigma, bullying, and body-image dissatisfaction, leading to anxiety, depressive symptoms, and reduced motivation to learn. Puhl and Lessard found that nearly two-thirds of adolescents with obesity report teasing or discrimination at school [6].

The educational environment itself can reinforce these effects. Teacher bias, whether conscious or implicit, may lead to lower expectations, fewer opportunities, and less positive reinforcement for students with obesity. According to self-determination theory, competence, autonomy, and relatedness are fundamental psychological needs; when these are undermined, intrinsic motivation declines, resulting in disengagement and poorer performance [10,37].

Behavioral patterns further contribute. Children with obesity often engage in lower levels of physical activity and higher levels of sedentary screen time, which both perpetuate obesity and limit opportunities for cognitive stimulation. Physical activity improves cerebral perfusion and neurotrophic factor release; Kang et al. [22] demonstrated that aerobic fitness partially mediates the link between BMI and academic outcomes.

Socioeconomic Determinants and Inequity

Across studies, SES, parental education, baseline cognitive ability, mental health, and comorbid conditions emerged as important confounders that may partly account for observed associations.

Studies consistently show that the obesity-achievement gap is amplified in lower socioeconomic groups. In deprived areas, unhealthy diets rich in inexpensive, energy-dense foods coexist with reduced access to safe play spaces and lower-quality schooling [5,22]. These “double burdens” compound disadvantage: poor nutrition affects brain development, while limited educational resources constrain cognitive growth. A further limitation of the evidence base is that the majority of included studies originate from high-income countries. Given that the steepest rises in childhood obesity are occurring in low- and middle-income countries, the generalizability of findings to these contexts remains uncertain.

At the school level, inequalities such as higher pupil-teacher ratios, fewer extracurricular opportunities, and smaller health budgets exacerbate this divide. Addressing obesity in isolation, without tackling the broader social determinants, risks perpetuating educational disparity. It is also important to recognize that pathways linking obesity and educational outcomes may differ between high-income and low-/middle-income countries due to variations in nutrition transitions, school resources, and social norms.

Cultural and Gender Dimensions

Cultural norms around body image and academic expectations shape both the experience and impact of obesity. Research from East Asia suggests that strong academic pressure may buffer educational outcomes despite obesity, whereas in Western contexts, appearance-based stigma exerts a stronger negative effect [23,24].

Gender differences are also notable; girls often internalize stigma more deeply, leading to anxiety-related academic difficulties, whereas boys’ underperformance is more strongly linked to reduced physical fitness and behavioral disengagement. Recognizing these patterns is vital for designing culturally sensitive interventions that are both effective and equitable.

Policy Implications

The findings underscore the need to embed health promotion into education policy. Frameworks such as the WHO Health-Promoting Schools initiative [10] and the CDC’s WSCC model [11] advocate holistic integration of physical activity, nutrition education, and emotional well-being into school curricula. 

The National Institute for Health and Care Excellence (NICE) guideline CG189 recommends early identification of at-risk children through school-based screening and family-centered counselling [27]. Similarly, the United Nations Children's Fund (UNICEF) Global Roadmap for Preventing Childhood Obesity calls for measures such as restricting unhealthy food advertising, improving school meal standards, and investing in safe recreational infrastructure [28]. Together, these frameworks position childhood obesity as both a health and educational justice issue.

Emerging digital health innovations, including wearable activity trackers, gamified exercise platforms, and well-being dashboards, offer novel opportunities to promote activity and engagement. However, robust ethical safeguards are essential to avoid surveillance or stigma. Data should be used to empower students and educators, not penalize them. Evidence supports the effectiveness of multi-component, whole-school approaches that combine high-quality nutrition provision, structured physical activity, and psychosocial support. Programs that engage families and address stigma appear to yield the most sustainable improvements in both well-being and academic engagement. For educators, policymakers, and public health practitioners, these findings highlight the importance of integrating physical activity, nutrition support, and psychosocial well-being into school environments to promote both learning and health.

Economic and Ethical Considerations

The economic rationale for integrating obesity prevention within education policy is strong. Poor academic performance correlates with reduced lifetime earnings and productivity, magnifying the societal cost of obesity beyond healthcare expenditure [27]. Interventions that enhance both learning and health deliver a dual return on investment.

Ethically, schools have a duty of care to foster environments promoting dignity and inclusion [38]. Anti-stigma policies, inclusive physical education programs, and teacher training on implicit bias are vital to ensure fairness. Preventing discrimination against children with obesity is both morally imperative and instrumental to improving educational participation.

Research Gaps and Future Directions

Despite a growing evidence base, key gaps remain. First, long-term longitudinal studies (>5 years) are scarce, limiting understanding of how early-life obesity trajectories shape later educational outcomes [19]. Second, neuroimaging and biochemical studies remain small and cross-sectional, necessitating larger cohorts integrating inflammatory markers, brain imaging, and neurocognitive data [8]. Third, most existing data derive from high-income countries, whereas the greatest rise in childhood obesity is occurring in low- and middle-income nations. Fourth, few interventions explicitly measure academic outcomes alongside BMI, limiting translational insight [25,26]. Future research would benefit from large-scale longitudinal cohorts, rigorously designed intervention trials, and cross-cultural comparisons to clarify causal pathways and improve global generalizability.

Interventions should adopt interdisciplinary, equity-focused designs, combining pediatric, educational, and behavioral sciences to elucidate mechanisms and identify scalable solutions. Furthermore, much of the evidence derives from observational studies, which restricts causal inference despite consistent associations. Overall, the existing literature is limited by heterogeneity in outcome measures, reliance on observational designs, short follow-up periods in interventional studies, and under-representation of low/middle-income countries (LMIC) settings. 

## Conclusions

This review synthesises a decade of evidence showing that childhood obesity is not only a medical condition but also an educational determinant. Across studies conducted in multiple countries and using diverse methodologies, a consistent pattern emerges: children with higher body mass index tend to achieve lower academic results than their normal-weight peers. This relationship persists even after accounting for socioeconomic factors, highlighting obesity’s independent and multifactorial impact on learning.

The mechanisms linking obesity and academic underachievement are complex and mutually reinforcing. Biologically, inflammation, hormonal dysregulation, and disturbed sleep affect neural circuits that support memory and executive function. Psychologically, weight stigma and social exclusion diminish self-confidence and concentration, while structural inequities such as poverty and limited access to healthy environments intensify disadvantage. Encouragingly, school-based interventions that combine physical activity, nutrition education, and emotional-wellbeing support improve both classroom participation and cognitive outcomes, even when weight change is modest. These findings challenge the notion that health and academic priorities compete; instead, they demonstrate that promoting physical wellbeing strengthens learning and engagement. Addressing childhood obesity therefore requires collaboration across health and education sectors. Schools should be recognised as vital health settings, and healthcare systems should consider educational progress as a marker of wellbeing. Policies that ensure affordable nutritious food, safe recreational spaces, and equitable resources for all schools are essential to breaking the cycle of inequality that links poor health and poor educational attainment.

Future research should focus on refining interventions and exploring causal mechanisms through longitudinal, interdisciplinary designs that integrate biological, cognitive, and psychosocial measures. These conclusions should be interpreted cautiously, as the evidence base remains largely observational and exploratory. Ultimately, tackling childhood obesity is not only a matter of improving physical health but of unlocking every child’s potential to learn, thrive, and participate fully in society.
